# The Long-Term Effects of a Kampo Medicine, Juzentaihoto, on Maintenance of Antibody Titer in Elderly People after Influenza Vaccination

**DOI:** 10.1155/2013/568074

**Published:** 2013-11-18

**Authors:** Ikuo Saiki, Keiichi Koizumi, Hirozo Goto, Akiko Inujima, Takao Namiki, Masaki Raimura, Toshiaki Kogure, Takeshi Tatsumi, Hiroki Inoue, Shinya Sakai, Hiroshi Oka, Makoto Fujimoto, Hiroaki Hikiami, Hiroaki Sakurai, Naotoshi Shibahara, Yutaka Shimada, Hideki Origasa

**Affiliations:** ^1^Division of Pathogenic Biochemistry, Institute of Natural Medicine, University of Toyama, Toyama 930-0194, Japan; ^2^Division of Kampo Diagnostics, Institute of Natural Medicine, University of Toyama, Toyama 930-0194, Japan; ^3^Department of Kampo Medicine, Hokusei Hospital, Toyama, Japan; ^4^Department of Japanese-Oriental (Kampo) Medicine, Graduate School of Medicine, Chiba University, Japan; ^5^AKIBA Clinic of Traditional Medicine, Chiba, Japan; ^6^Department of Japanese Oriental Medicine, Gunma Central & General Hospital, Gunma, Japan; ^7^Department of Internal Medicine, Ninosawa Hospital, Gunma, Japan; ^8^Department of Japanese Oriental (Kampo) Medicine, Iizuka Hospital, Fukuoka, Japan; ^9^Tonami Miwa Hospital, Toyama, Japan; ^10^Department of Kampo Medicine, Bayside Clinic, Kanagawa, Japan; ^11^Department of Japanese-Oriental (Kampo) Medicine, Graduate School of Medicine and Pharmaceutical Sciences, University of Toyama, Japan; ^12^Department of Cancer Cell Biology, Graduate School of Medicine and Pharmaceutical Sciences, University of Toyama, Japan; ^13^Department of Biostatistics and Clinical Epidemiology, Graduate School of Medicine and Pharmaceutical Sciences, University of Toyama, Japan

## Abstract

We have performed a broad-ranging analysis of the adjuvant effect of a Kampo medicine, juzentaihoto (JTT), on influenza vaccination in a multicenter randomized controlled trial. In this study, the enhancing effect of JTT on antibody titer after influenza vaccination was studied for 28 weeks in elderly people who were in the high-risk group for influenza infection. In total, 91 subjects over 65 years old were recruited from four long-term-care facilities located in Chiba, Gunma, and Toyama prefectures in Japan. Participants were randomly assigned to the JTT and the control groups. Blood samples were taken at 4 weeks before vaccination, at the time of vaccination, and then at 4, 8, 12, and 24 weeks after vaccination. The hemagglutination inhibition (HI) titers against A/California/7/2009 (H1N1), A/Victoria/210/2009 (H3N2), and B/Brisbane/60/2008 were then manually measured. A significant increase in HI titer against H3N2 was observed at week 8 after vaccination in the JTT group compared with the control group (*P* = 0.0229), and the HI titer of the JTT group significantly increased from 4 to 24 weeks (*P* = 0.0468), compared with the control group. In conclusion, our results indicated that JTT increased and prolonged antibody production against A/Victoria/210/2009 (H3N2), in particular, after influenza vaccination.

## 1. Introduction

Influenza vaccination is an important procedure for protecting against influenza infection. Vaccine adjuvants that induce the specific immune responses against the vaccine antigens are valuable for enhancing the efficacy of influenza vaccines by increasing and maintaining antibody production.

Some Kampo formulas, such as juzentaihoto (JTT), have been reported to show nonspecific preventive effects against influenza infection [1]. Some basic studies have shown that JTT enhanced immune cell function such as natural killer cell activity [2], cytokine production [3], and immunoadjuvant activity against human papillomavirus [4].

In the present study, we investigated the adjuvant effect of long-term administration of JTT on influenza vaccination in a randomized controlled trial among elderly people who may be in the high-risk group for influenza infection.

## 2. Materials and Methods

### 2.1. Participants

Subjects over 65 years old were recruited from four long-term-care facilities (Akiba Clinic of Traditional Medicine in Chiba Prefecture, Ninosawa Hospital in Gunma Prefecture, Tonami Miwa Hospital and Sanbari Takaoka Hospital in Toyama Prefecture) in Japan. Patients with severe infection, neoplastic disease, or poor general condition were excluded. At baseline, each patient underwent a uniform evaluation that included medical history, principal disease, main complications, and the degree of nursing care.

**Figure 1:**
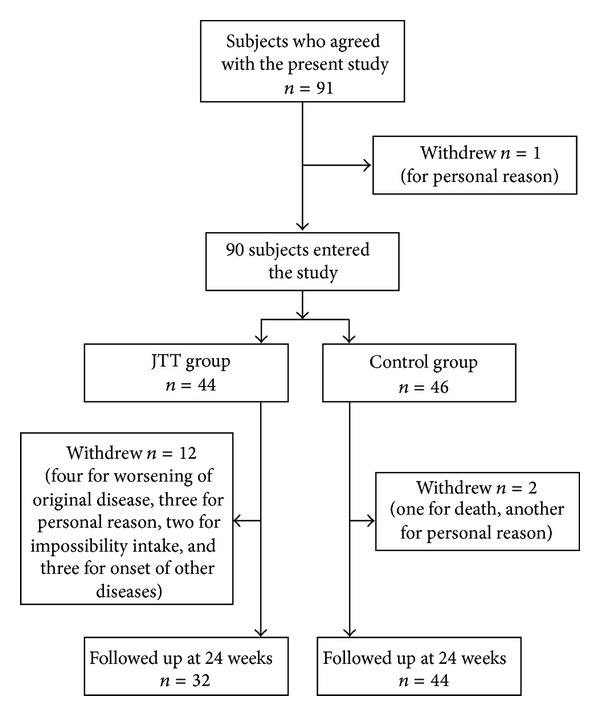
Flow chart of subject recruitment and trial profiles.

A total of 91 patients (22 men and 69 women: mean age ± SD, 85.6 ± 8.1 years) were enrolled, but one patient withdrew before the allocation, leaving a total of 90 participants. These patients were randomly allocated into either the JTT group (*n* = 44) or the control group (*n* = 46) (Figure 1). No significant differences were noted between the two groups in terms of age (85.6 ± 8.2 and 85.5 ± 8.3 year, resp.) or duration of hospitalization (35.6 ± 29.8 and 37.9 ± 35.5 months, resp.). Original diseases in these patients included cerebral vascular disease in 54 patients, dementia in 37, and bone and joint diseases in 14. Regarding the ranks of care, 8 patients were in Rank 1, 7 patients were in Rank 2, 10 patients were in Rank 3, 17 patients were in Rank 4, and 48 patients were in Rank 5. Almost all of them were bedridden patients (Table 1). HI titer was measured twice before vaccination (−4 week and 0 week). As a result, both HI in JTT and control group were guaranteed to be the same level statistically. The study design was approved by the Human Subjects Committee, University of Toyama. All patients provided written informed consent in accordance with ethical guidelines set forth in the 1975 Declaration of Helsinki.

### 2.2. Intervention Protocol

A 28-week randomized controlled trial was begun between October 2011 and April 2012. Participants were assigned to the JTT and control groups using the allocation software managed by bystander. The extract of JTT, which is approved for medical use in Japan, was purchased from Kracie Holdings, Ltd. (Tokyo, Japan). It consists of ten herbs: 3 g each of Astragali radix (roots of *Astragalus membranaceus* Bunge), Cinnamomi cortex (bark of *Cinnamomum cassia* Blume), Rehmanniae radix (root of *Rehmannia glutinosa* Libosch var. *purpurea* Makino), Paeonia radix (rhizome of *Paeonia lactiflora* Pall), Cnidii rhizoma (rhizome of *Cnidium officinale* Makino), Atractylodis lanceae rhizoma (rhizome of *Atractylodes lancea* DC), Angelicae radix (root of *Angelica acutiloba* Kitagawa), Ginseng radix (root of *Panax ginseng* C. A. Meyer), Hoelen (fungus of *Poria cocos* Wolf), and 1.5 g of Glycyrrhizae radix (root of *Glycyrrhiza uralensis* Fisch et DC). The aqueous extract was lyophilized to obtain a powder. Fine grains (7.5 g) consist of lactose (1.3 g) and the aqueous extract (6.2 g).

The quality of the JTT extract was confirmed by its 3D HPLC pattern [5]. Subjects in the JTT group were administered JTT at 7.5 g/day, 3.75 g two times daily, orally or through a tube after meals, from 4 weeks before to 24 weeks after vaccination. Those in the control group were not administered Kampo medication but continued to take their regular medication. The premixed vaccine contained 15 *μ*g of each of following hemagglutinin (HA) antigens: A/California/7/2009 (H1N1), A/Victoria/210/2009 (H3N2), and B/Brisbane/60/2008. A subcutaneous injection of 0.5 mL of vaccine was given once at week 0. This study was conducted as an open-label study since it was impossible to prepare a suitable placebo due to the unique flavor and odor of JTT.

### 2.3. Outcome Determination

Blood samples were taken at weeks −4, 0, 4, 8, 12, and 24. Serum samples were kept frozen at −80°C until analysis. Antibody titers to hemagglutinin were measured by a standard microtiter hemagglutination inhibition method [6]. Blood biochemistry was measured by standard methods as follows: albumin (Alb) was measured by a BCG method. Aspartate aminotransferase (AST), alanine aminotransferase (ALT), lactate dehydrogenase (LDH), and blood urea nitrogen (BUN) were measured by a UV method. Alkaline phosphatase (ALP) was measured by a colorimetric method. Creatinine (Cr) was measured by an enzyme method. Natrium (Na), potassium (K), and chloride (Cl) were measured by an electrode method.

### 2.4. Statistical Analysis

Data are expressed as means ± SD. Blood biochemistry data of the two groups were compared at weeks −4, 0, 4, 8, 12, and 24 with the Student's *t*-test.

HI titers of the two groups were compared at weeks 4, 8, 12, and 24 with the Cochran-Armitage trend test. The two groups were compared from 4 to 24 weeks by the nonparametric repeated analysis of variance (ANOVA) where HI titers had been transformed into ordinal data. A *P* value < 0.05 was considered statistically significant. The nonparametric version means that the titer data were transformed into ordinal data such as “less than 10 times” into “0”; “10 times” into “1”; “20 times” into “2”; “40 times” into “3”; “80 times” into “4”; “160 times” into “5”; “320 times” into “6”; “640 times” into “7”; and “1280 times” into “8” on this occasion. The reason of transforming the data was that original titer data were strongly right skewed. A *P* value < 0.05 was considered to indicate statistical significance.

## 3. Results

### 3.1. Characteristics of Participants

Twelve JTT subjects dropped out of the study due to primary disease progression (*n* = 4), revocation of agreement and drug refusal (*n* = 3), impossibility of oral intake before starting the study (*n* = 2), ileus (*n* = 1), cerebral infarction (*n* = 1), or stomach distress (*n* = 1). Two control subjects also dropped out due to primary disease progression (*n* = 1) and revocation of agreement (*n* = 1). No subject contracted influenza during the study period. Data were analyzed with the remaining 76 subjects (32 and 44 in the JTT and control groups, resp.). Throughout the study period, obvious adverse events with JTT were noted in one subject, who developed epigastric distress and stopped taking JTT. Thereafter, his symptoms disappeared. JTT contains *Glycyrrhiza*, which may induce hypokalemia, but no subjects showed decreased serum potassium in this study. No other major blood biochemistry changes occurred before and after the study in the JTT and control groups statistically (Table 2).

### 3.2. Comparison of the HI Titers of the JTT and Control Groups at Weeks −4, 0, 4, 8, 12, and 24

The number of group members who had high HI titers for A/Victoria/210/2009 (H3N2) tended to increase in the JTT group compared with the control group at weeks 4, 12, and 24 (*P* = 0.0739, *P* = 0.0849, and *P* = 0.0895, resp.). The number of group members with a high HI titer significantly increased in the JTT group compared to the control group at week 8 (*P* = 0.0229). In contrast, no significant difference was noted between the two groups for the titers for A/California/7/2009 (H1N1) or B/Brisbane/60/2008 in any week (Table 3). One sample at 24 weeks in the JTT group could not be measured for lack of volume. Thus, the number of samples at 24 weeks in the JTT group was 31 samples.

### 3.3. Comparison of the Changes of HI Titer for H3N2 from −4 to 24 Weeks in the JTT and Control Groups

**Figure 2:**
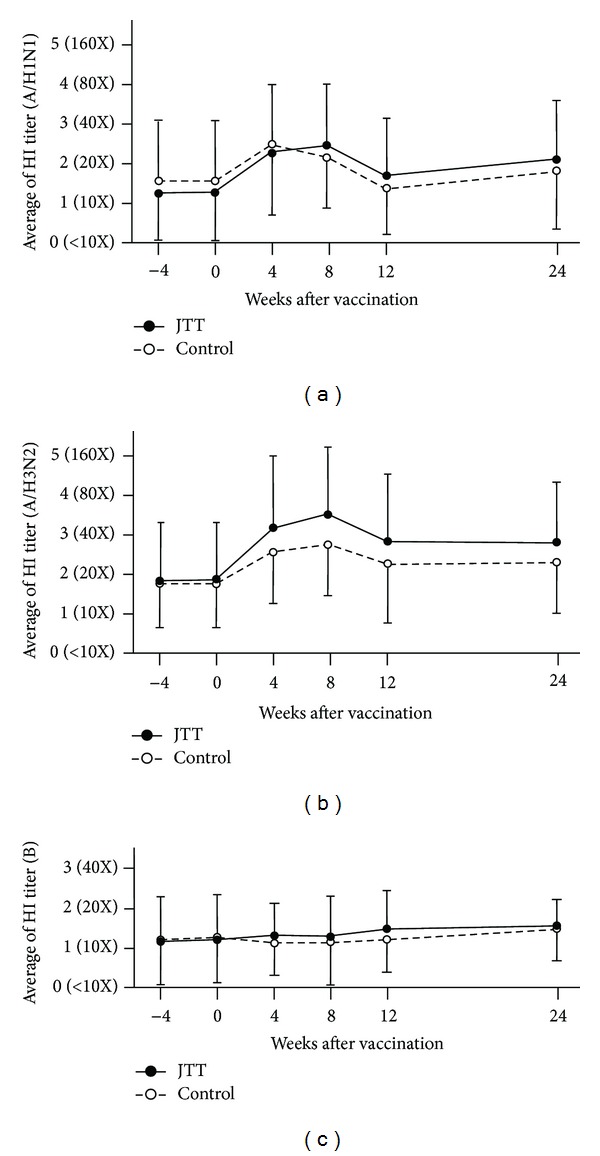
Changes in HI titer after vaccination. (a) A/California/7/2009 (H1N1), (b) A/Victoria/210/2009 (H3N2), and (c) B/Brisbane/60/2008. The HI titer significantly increased for A/Victoria/210/2009 (H3N2) in the JTT group compared to the control group from 4 to 24 weeks after JTT administration and vaccination (mean ± SD, *P* = 0.0468).

The changes in HI titer for H3N2 significantly increased in the JTT group compared with the control group from 4 to 24 weeks (*P* = 0.0468). However, no significant difference was noted between the two groups for H1N1 or B (Figure 2).

## 4. Discussion

This study was designed to examine the adjuvant effect of JTT on the administration of influenza vaccine in a long-term study in elderly people using a multicenter randomized controlled trial with the goal of increasing the effects of antibody production. Overall, the number of patients with a high HI titer tended to increase in the JTT group more than in the control group at 4 weeks and was significantly increased at 8 weeks for A/Victoria/210/2009 (H3N2). This tendency toward an increase continued until 24 weeks.

This result makes it clear that there is a significant difference between the JTT group and the control group on the time course of their HI titers of H3N2 by ANOVA. These results showed that JTT had an adjuvant effect especially for the A/Victoria/210/2009 (H3N2) vaccine.

Because the HI titer was measured twice before vaccination (−4 weeks and 0 weeks), HI in both the JTT group and the control group was guaranteed to be at the same level statistically. Therefore, the assignment of participants is thought to be no issue.

It is generally known that the H1N1 influenza A virus caused the catastrophic and historic pandemic in 1918-1919, and that the 1968-1969 pandemic was caused by the H3N2 influenza A virus. However, the genetic background of the present seasonal H1N1 and H3N2 viruses has not changed from the pandemic H1N1 of 1918-1919 and the pandemic H3N2 of 1968-1969, respectively [7]. Taking the average age of the 91 participants (85.6 years) into consideration, it is very likely that the participants were infected with pandemic H3N2 in 1968-1969, not but with pandemic H1N1 in 1918-1919. Therefore, the present seasonal H3N2 influenza vaccine might strongly affect the immunoresponse in elderly participants because this vaccine was prepared from the seasonal H3N2 influenza virus, which is the same as the pandemic H3N2 from 1968-1969.

In addition, the possibility cannot be denied that the present seasonal H3N2 influenza vaccine has high immunogenicity. In fact, two previous randomized trials have reported on antibody production after influenza vaccination with Kampo medicines. The first one was a trial using maobushisaishinto (MBST), which is often used to treat bronchitis patients. The mean HI titer against H3N2 significantly increased in the group taking MBST for three weeks before or after influenza vaccination compared to the control group, at 4 weeks after influenza vaccination [8]. However, no prolonged effect of MBST was reported because no observations were made over time.

On the other hand, the second was a trial on the effect of hochuekkito (HET), which, like JTT, is frequently used to improve chronic fatigue. In this study, healthy adults were administered HET orally for two weeks before influenza vaccination, and then the variability of the mean HI titer was measured from week 0 to week 12 after influenza vaccination. No discernible difference was noted in the antibody production between the HET group and the control group [9]. One explanation for this negative result may be that this study was designed for healthy adults whose ability for antibody production did not decrease, unlike the case for elderly people, and the subjects maintained a normal titer level. Basic research on HET has indicated an ability to increase IgA antibody production against a model vaccine antigen [10]. In a clinical study, administration of HET potentiated the natural killer cell activity in elderly people [11]. These results suggest that the adjuvant effects of Kampo medicines on influenza vaccination should be appropriately determined based on the types of Kampo medicine, the length of treatment, and the types of patients, and so forth.

Clinically, JTT has been used to improve conditions such as chronic fatigue, postoperative decline, anorexia, and anemia. It has also shown effectiveness in treating various symptoms and restoring strength in elderly people and has proven suitable for long-term administration [12]. Some clinical reports on JTT have shown hematopoiesis and anemia recovery [13–15]. JTT has been used as an adjunctive therapy for advanced breast cancer patients [16]. A recent report indicated that JTT aided in preventing the recurrence of otitis [17].

JTT is prescribed for various diseases, and one of its mechanisms of action is to augment the immune system through anticancer effects due to immunopotentiation [3]. Some studies have reported that JTT stimulates antibody production in tumor-bearing mice [18, 19] and that the pectic polysaccharide contained in JTT is partly associated with this increase in antibody production [20]. JTT has been also shown to act as a vaccine adjuvant to stimulate an immune response against human papillomavirus in mice [4]. However, clinical randomized controlled trials of JTT and antibody production have not yet been carried out.

The present study indicates that JTT is a useful Kampo formulation to augment the efficacy of influenza vaccination of elderly people/patients whose ability to produce antibodies may be impaired. Although no severe side effects have been reported for JTT [14, 17, 21]. In the early stage, many patients dropped out of JTT group in comparison with control group. One of the reasons may be that some elderly people rejected oral administration of JTT because they disliked its taste.

Furthermore, four patients whose preexisting diseases worsened became decreasingly able to take JTT orally and eventually stopped. Meanwhile, because they did not take a placebo in this study, there were few patients in the control group who dropped out. In the present study, a direct adverse effect of JTT was indicated by the one case in which there was a complaint of a heavy feeling in the stomach.

In the present study, a direct adverse effect of JTT was indicated by the one case who complained of a heavy feeling in the stomach.

Finally, further investigation of the active components in JTT is necessary to augment the influenza vaccination as well as to establish its mechanisms of action. The optimal period for intake and suitable conditions for subjects also require clinical study.

## 5. Conclusion

The adjuvant effects of JTT on influenza vaccination were studied in elderly patients in long-term-care facilities. JTT increased and prolonged antibody production against A/Victoria/210/2009 (H3N2) after influenza vaccination at 8 weeks.

## Figures and Tables

**Table 1:** Clinical and demographic characteristics of study participants.

Group	JTT group	Control group
Sex (male/female)	10/34	11/35
Age (year), mean ± SD	85.6 ± 8.2	85.5 ± 8.3
Duration of hospitalization (month), mean ± SD	35.6 ± 29.8	37.9 ± 35.5
Chief disease (number)		
Cerebral vascular disorder	26	28
Dementia	19	18
Bone and joint disease	6	8
Rank of care (number)		
Rank 1	3	5
Rank 2	3	4
Rank 3	6	4
Rank 4	9	8
Rank 5	23	25

*Note*. All group comparisons *P* > 0.05. SD: standard deviation; JTT: Juzentaihoto.

Rank 1: patients who need partial help with personal care. Rank 2: patients who need partial help with eating or going to the toilet and so forth. Rank 3: patients who need full help with certain activities of daily living. Rank 4: patients who have difficulty performing all daily activities without help. Rank 5: patients who find it impossible to perform all daily activities without help.

**Table 2:** Changes in blood biochemistry data of JTT and control groups.

	Group	−4 weeks	0 week	4 weeks	8 weeks	12 weeks	24 weeks
Alb (g/dL)	JTT (*n* = 42–32)	3.6 ± 0.37	3.5 ± 0.28	3.6 ± 0.31	3.7 ± 0.32	3.6 ± 0.31	3.4 ± 0.49
	Cont (*n* = 45–44)	3.6 ± 0.43	3.6 ± 0.42	3.7 ± 0.45	3.6 ± 0.40	3.7 ± 0.47	3.6 ± 0.42
AST (IU/L)	JTT (*n* = 42–32)	23.4 ± 10.2	22.9 ± 10.8	24.0 ± 11.9	24.8 ± 10.8	26.1 ± 17.7	24.6 ± 10.0
	Cont (*n* = 45–44)	22.0 ± 7.2	25.0 ± 12.0	23.9 ± 9.5	23.0 ± 7.4	23.2 ± 7.0	23.4 ± 7.4
ALT (IU/L)	JTT (*n* = 42–32)	19.9 ± 13.4	18.1 ± 13.0	18.3 ± 12.7	19.9 ± 15.0	20.5 ± 17.2	20.5 ± 15.3
	Cont (*n* = 45–44)	18.1 ± 9.5	20.7 ± 14.6	18.8 ± 12.3	18.0 ± 10.0	17.3 ± 9.0	17.9 ± 9.0
LDH (IU/L)	JTT (*n* = 42–32)	168.0 ± 30.1	175.8 ± 33.1	179.5 ± 47.2	180.3 ± 43.1	180.4 ± 43.3	194.9 ± 55.0
	Cont (*n* = 45–44)	177.8 ± 39.0	186.7 ± 50.3	179.2 ± 43.9	176.7 ± 38.3	183.7 ± 41.4	185.2 ± 41.2
ALP (IU/L)	JTT (*n* = 42–32)	379.4 ± 465.0	363.8 ± 432.7	379.7 ± 454.4	379.1 ± 402.7	398.6 ± 461.7	404.8 ± 385.7
	Cont (*n* = 45–44)	278.3 ± 62.8	280.0 ± 66.7	293.6 ± 82.2	289.9 ± 71.0	297.2 ± 75.6	319.3 ± 94.4
BUN (mg/dL)	JTT (*n* = 42–32)	21.1 ± 9.7	19.0 ± 8.7	19.9 ± 8.9	20.7 ± 9.5	20.0 ± 8.6	21.5 ± 10.6
	Cont (*n* = 45–44)	17.6 ± 5.6	16.9 ± 6.3	18.1 ± 5.9	17.0 ± 6.1	17.2 ± 5.4	18.1 ± 5.3
Cr (mg/dL)	JTT (*n* = 42–32)	0.82 ± 0.45	0.70 ± 0.36	0.68 ± 0.36	0.68 ± 0.34	0.69 ± 0.36	0.71 ± 0.42
	Cont (*n* = 45–44)	0.70 ± 0.22	0.64 ± 0.20	0.64 ± 0.20	0.64 ± 0.21	0.64 ± 0.20	0.64 ± 0.19
Na (mEq/L)	JTT (*n* = 42–32)	138.1 ± 5.8	138.1 ± 5.0	139.7 ± 5.1	138.8 ± 4.8	139.3 ± 4.9	139.0 ± 5.1
	Cont (*n* = 45–44)	138.6 ± 4.2	137.5 ± 4.8	139.0 ± 4.6	137.6 ± 3.9	138.6 ± 3.6	138.6 ± 3.6
K (mEq/L)	JTT (*n* = 42–32)	4.17 ± 0.50	4.10 ± 0.55	4.09 ± 0.62	4.46 ± 0.59	4.30 ± 0.51	4.23 ± 0.67
	Cont (*n* = 45–44)	3.88 ± 0.43	4.05 ± 0.45	4.18 ± 0.44	4.65 ± 0.74	4.42 ± 0.62	4.41 ± 0.69
Cl (mEq/L)	JTT (*n* = 42–32)	99.8 ± 5.7	100.0 ± 4.7	100.6 ± 4.1	100.1 ± 4.7	100.7 ± 4.6	99.3 ± 5.2
	Cont (*n* = 45–44)	100.0 ± 4.2	99.4 ± 5.0	100.3 ± 4.2	100.0 ± 4.2	100.3 ± 3.7	99.7 ± 3.5

JTT: Juzentaihoto group; Cont: control group; values are means ± SD.

**Table 3:** Comparison in number of the JTT group and the control group on every HI titer at weeks −4, 0, 4, 8, 12, and 24.

	Number of every HI titer (times)									
	<10	10	20	40	80	160	320	640	1280	*P* value
A/California/7/2009 (H1N1)										
−4 weeks										
JTT (*n* = 42)	13	12	10	2	4	1	0	0	0
Cont (*n* = 45)	14	11	5	11	4	0	0	0	0
0 week										
JTT (*n* = 37)	12	10	9	2	3	1	0	0	0
Cont (*n* = 45)	13	12	5	11	4	0	0	0	0
4 weeks										
JTT (*n* = 36)	3	10	7	7	7	0	2	0	0	0.8235
Cont (*n* = 44)	2	10	12	10	7	3	0	0	0	
8 weeks										
JTT (*n* = 34)	2	9	8	7	4	2	2	0	0	0.3529
Cont (*n* = 44)	6	11	7	10	10	0	0	0	0	
12 weeks										
JTT (*n* = 33)	8	10	6	4	3	2	0	0	0	0.4028
Cont (*n* = 45)	11	16	6	11	1	0	0	0	0	
24 weeks										
JTT (*n* = 31)	3	8	10	4	4	2	0	0	0	0.2848
Cont (*n* = 44)	9	12	5	15	3	0	0	0	0	

A/Victoria/210/2009 (H3N2)										
−4 weeks										
JTT (*n* = 42)	8	11	10	6	4	3	0	0	0
Cont (*n* = 45)	4	15	15	6	4	1	0	0	0
0 week										
JTT (*n* = 37)	6	11	9	4	4	3	0	0	0
Cont (*n* = 45)	4	14	16	6	4	1	0	0	0
4 weeks										
JTT (*n* = 36)	1	8	5	5	8	6	2	0	1	0.0739
Cont (*n* = 44)	1	10	9	18	3	1	1	1	0	
8 weeks										
JTT (*n* = 34)	0	5	6	7	6	5	4	0	1	0.0229
Cont (*n* = 44)	0	7	14	15	5	1	1	0	1	
12 weeks										
JTT (*n* = 33)	1	8	5	7	7	3	1	0	1	0.0849
Cont (*n* = 45)	2	15	8	14	4	0	1	0	1	
24 weeks										
JTT (*n* = 31)	0	8	5	8	6	2	1	0	1	0.0895
Cont (*n* = 44)	1	13	12	12	4	1	0	0	1	

B/Brisbane/60/2008										
−4 weeks										
JTT (*n* = 42)	12	17	5	6	2	0	0	0	0
Cont (*n* = 45)	10	23	4	7	0	1	0	0	0
0 week										
JTT (*n* = 37)	11	15	5	4	2	0	0	0	0
Cont (*n* = 45)	10	20	8	6	0	1	0	0	0
4 weeks										
JTT (*n* = 36)	4	19	10	3	0	0	0	0	0	0.2576
Cont (*n* = 44)	9	21	13	1	0	0	0	0	0	
8 weeks										
JTT (*n* = 34)	7	15	8	3	1	0	0	0	0	0.4411
Cont (*n* = 44)	13	19	8	3	0	1	0	0	0	
12 weeks										
JTT (*n* = 33)	3	17	8	4	1	0	0	0	0	0.2610
Cont (*n* = 45)	3	32	6	3	1	0	0	0	0	
24 weeks										
JTT (*n* = 31)	1	16	13	1	0	0	0	0	0	0.6710
Cont (*n* = 44)	1	28	13	1	1	0	0	0	0	
